# High expression of RNF31 is associated with tumor immune cell infiltration and leads to poor prognosis in liver hepatocellular carcinoma

**DOI:** 10.1038/s41598-023-32692-4

**Published:** 2023-04-28

**Authors:** Guifu Xi, Runfen Cheng, Leiting Liang, Na Che, Yalei Wang, Nan Zhao, Xiaohui Liang, Bing Shao, Xiulan Zhao, Danfang Zhang

**Affiliations:** 1 Department of Pathology, Tianjin Medical University, Tianjin, 300070 China; 2 National Clinical Research Center for Cancer, Key Laboratory of Cancer Prevention and Therapy, Tianjin’s Clinical Research Center for Cancer, Tianjin Medical University Cancer Institute and Hospital, Tianjin, 300060 China

**Keywords:** Cancer, Computational biology and bioinformatics, Immunology, Biomarkers, Oncology

## Abstract

Ring finger protein 31 (RNF31) has been found to play an important role in tumor immunity. However, the role of RNF31 in liver hepatocellular carcinoma (LIHC) has not been reported. Therefore, we investigated the expression and prognostic value of RNF31 in patients with LIHC and explored its relationship with immune cell infiltration. The Cancer Genome Atlas liver hepatocellular carcinoma (TCGA-LIHC) dataset was downloaded to analyse the impact of RNF31 on the prognosis and immune cell infiltration of LIHC. The Tumor Immune Estimation Resource (TIMER) database was used to analyse the correlation between RNF31 and tumor immune cell infiltration in LIHC. Additionally, we analysed the relationship between RNF31 and tumor necrosis factor (TNF) as well as the interferon-gamma (IFN-γ) signaling pathway. The expression of RNF31 in LIHC was significantly higher than that in normal tissues. Increased RNF31 expression was associated with decreased overall survival (OS) and relapse-free survival (RFS). An increase in RNF31 expression was closely related to the infiltration levels of immune cells (e.g., natural killer (NK) cells, CD8 + T cells, and B cells). RNF31 was also positively correlated with the expression of immune checkpoint genes in LIHC. Moreover, RNF31 may participate in TNF and IFN-γ signaling pathways. In conclusion, RNF31 is a potentially valuable prognostic biomarker in LIHC. RNF31 is also associated with immune cell infiltration in LIHC. RNF31 may be a potential target for immunotherapy of LIHC.

## Introduction

LIHC, accounting for nearly 90% of all primary liver cancers, is one of the most common malignancies worldwide and the third leading cause of cancer-related deaths^1,2^. In China, liver cancer is also among the cancers with the worst prognosis^3^. This global problem can lead to more than 700,000 deaths each year^4^. At present, the overall prognosis of LIHC is not ideal. It is difficult to treat because it is usually in an advanced stage at the time of diagnosis and the 5-year survival rate is less than 12%^5,6^. In addition, the high recurrence rate of most patients with LIHC also leads to a poor prognosis^7,8^. In recent years, the treatment of liver cancer has made rapid progress. The discovery of new biomarkers and the promotion of liquid biopsy technology have greatly enhanced the early diagnosis and treatment of LIHC^9^. With the emergence of various advanced therapies, immunotherapy has gradually become an important treatment for liver cancer^10–12^. The progression of LIHC is regulated by the immune system. Systemic therapies are used for advanced disease. However, until 2017, only antiangiogenic tyrosine kinase inhibitors (TKIs) were included. Immune checkpoint inhibitors have shown strong antitumor activity in some patients. The anti-PD1 (PD1: programmed cell death protein 1) drugs nivolumab and pembrolizumab are used after TKIs in many cases^13^. Some recent evidence suggests that one type of immunotherapy alone is ineffective for cancer, including LIHC^12^. Effective combinations of immunotherapy are therefore needed. Finding predictive biomarkers and more effective combinations or sequential therapies is a major challenge for the treatment of LIHC.

RNF31 has been shown to play an important role in tumor immunity. RNF31, an E3 ubiquitin ligase, is one of the three important components of the linear ubiquitination chain assembly complex (LUBAC). Inhibition of RNF31 (either genetically or pharmacologically) greatly increases the sensitivity of melanoma, breast cancer, and colorectal cancer cells to NK and CD8 + T cells. This immune clearance effect occurs in a TNF-dependent manner, enhancing the immune bystander response^14^. RNF31 deletion sensitizes pancreatic cancer to T-cell-mediated cytotoxicity^15^. These results suggest that RNF31 may be a prognostic biomarker and therapeutic target for cancers.

However, no study has clarified the role of RNF31 in LIHC. In this study, we analysed the expression of RNF31 between LIHC and normal tissues. We then analysed the effect of RNF31 on OS and RFS. We also studied the relationship between RNF31 and immune checkpoint genes, infiltrating immune cells, and immune-related pathway activity in LIHC. We believe a better understanding of RNF31 in LIHC may contribute to the further development of cancer immunotherapy.

## Results

### RNF31 expression is different between tumor and normal tissues across human cancers

RNF31 has been found to be overexpressed and exhibit primarily oncogenic roles in several cancers. In order to compare the effects of RNF31 on LIHC and other cancers. We first analysed the expression of RNF31 in multiple human cancers and the relationship between RNF31 and immune cells using public databases.

**Figure 1:**
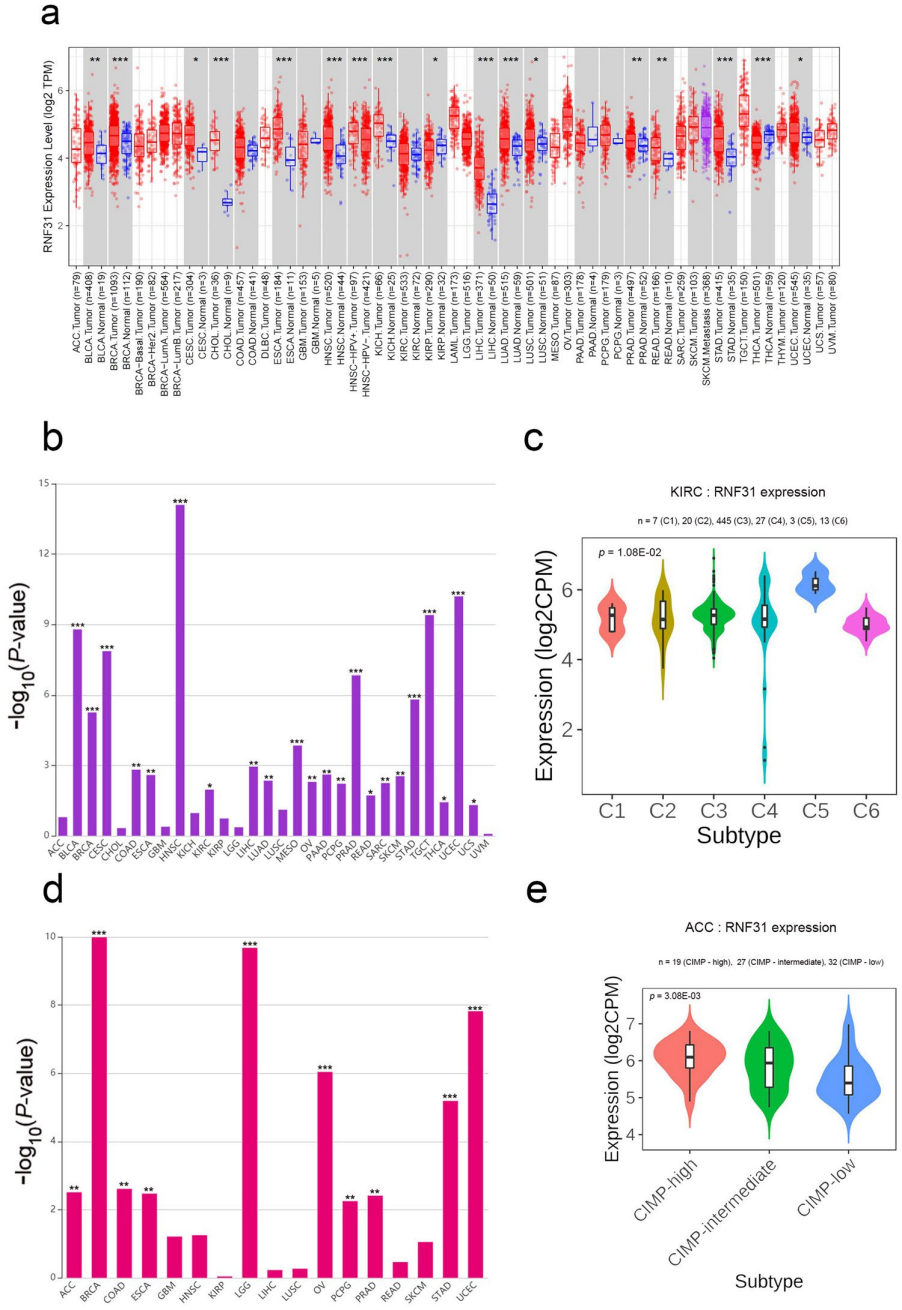
RNF31 expression levels in human cancers. (**a**) Detection of the expression level of RNF31 in different cancers in TCGA database by TIMER (TPM = transcripts per million, *P < 0.05, **P < 0.01, ***P < 0.001). (**b**) Kruskal–Wallis Test results of RNF31 expression in different immune subtypes across human cancers (*P < 0.05, **P < 0.01, ***P < 0.001). (**c**) Expression levels of RNF31 in different immune subtypes of KIRC (n = number of patients with available data, log2CPM: log2 on counts per millions). (**d**) Kruskal–Wallis Test results of RNF31 expression in different molecular subtypes across human cancers (*P < 0.05, **P < 0.01, ***P < 0.001). (**e**) Expression levels of RNF31 in different molecular subtypes of ACC. CIMP: CpG island methylation phenotype, ACC: adrenocortical carcinoma, BLCA: bladder urothelial carcinoma, BRCA: breast invasive carcinoma, CESC: cervical and endocervical cancers, CHOL: cholangiocarcinoma, COAD: colon adenocarcinoma, DLBC: lymphoid neoplasm diffuse large B-cell lymphoma, ESCA: esophageal carcinoma, GBM: glioblastoma multiforme, HNSC: head and neck squamous cell carcinoma, KICH: kidney chromophobe, KIRC: kidney renal clear cell carcinoma, KIRP: kidney renal papillary cell carcinoma, LAML: acute myeloid leukemia , LGG: brain lower grade glioma, LIHC: liver hepatocellular carcinoma, LUAD: lung adenocarcinoma, LUSC: lung squamous cell carcinoma, MESO: mesothelioma, OV: ovarian serous cystadenocarcinoma, PAAD: pancreatic adenocarcinoma, PCPG: pheochromocytoma and paraganglioma, PRAD: prostate adenocarcinoma, READ: rectum adenocarcinoma, SARC: sarcoma, SKCM: skin cutaneous melanoma, STAD: stomach adenocarcinoma, TGCT: testicular germ cell tumors, THCA: thyroid carcinoma, THYM: thymoma, UCEC: uterine corpus endometrial carcinoma, UCS: uterine carcinosarcoma, UVM: uveal melanoma.

We primarily analysed the gene expression levels of RNF31 in multiple tumors and normal tissues based on the TIMER database. Among them, RNF31 was highly expressed in 15 types of cancer (including bladder urothelial carcinoma (BLCA), breast invasive carcinoma (BRCA), cervical and endocervical cancers (CESC), cholangiocarcinoma (CHOL), colon adenocarcinoma (COAD), esophageal carcinoma (ESCA), head and neck squamous cell carcinoma (HNSC), kidney chromophobe (KICH), LIHC, lung adenocarcinoma (LUAD), lung squamous cell carcinoma (LUSC), prostate adenocarcinoma (PRAD), rectum adenocarcinoma (READ), stomach adenocarcinoma (STAD), and uterine corpus endometrial carcinoma (UCEC)). In contrast, in kidney renal papillary cell carcinoma (KIRP) and thyroid carcinoma (THCA), RNF31 expression levels were lower than those in normal controls (Fig. 1a). These results suggest that RNF31 mRNA expression is significantly increased in most tumor types.

Next, we explored the relationship between RNF31 expression and immune and molecular subtypes across human cancers. Immune subtypes are classified into six types, including C1 (wound healing), C2 (IFN-gamma dominant), C3 (inflammatory), C4 (lymphocyte depleted), C5 (immunologically quiet) and C6 (TGF-βdominant, TGF-β: transforming growth factor-beta). We performed the Kruskal–Wallis test to analyse RNF31 expression in different immune subtypes across human cancers from TISIDB (Fig. 1b). RNF31 expression was related to different immune subtypes in BLCA, BRCA, CESC, COAD, ESCA, HNSC, kidney renal clear cell carcinoma (KIRC), LIHC, LUAD, mesothelioma (MESO), ovarian serous cystadenocarcinoma (OV), pancreatic adenocarcinoma (PAAD), pheochromocytoma and paraganglioma (PCPG), PRAD, READ, sarcoma (SARC), skin cutaneous melanoma (SKCM), STAD, testicular germ cell tumors (TGCT), THCA, UCEC and uterine carcinosarcoma (UCS). Additionally, the expression of RNF31 varied in different tumor immune subtypes. For example, in KIRC, RNF31 was highly expressed in type C5 and expressed at low levels in type C6 (Fig. 1c). We also performed the Kruskal–Wallis test to analyse RNF31 expression in different molecular subtypes across human cancers from TISIDB (Fig. 1d). We found a significant association between RNF31 expression and different molecular subtypes of adrenocortical carcinoma (ACC), BRCA, COAD, ESCA, brain lower grade glioma (LGG), OV, PCPG, PRAD, STAD and UCEC. The expression of RNF31 varied among different tumor molecular subtypes. For example, the RNF31 expression level was higher in the CIMP-high (CIMP: CpG island methylation phenotype) subtype than in the other two subtypes in ACC (Fig. 1e). Based on these results, we conclude that the expression of RNF31 is different in different immune and molecular subtypes of human tumors.

### Analysis of the correlation of RNF31 with immune cell infiltration levels and immune marker gene expression across cancers

**Figure 2:**
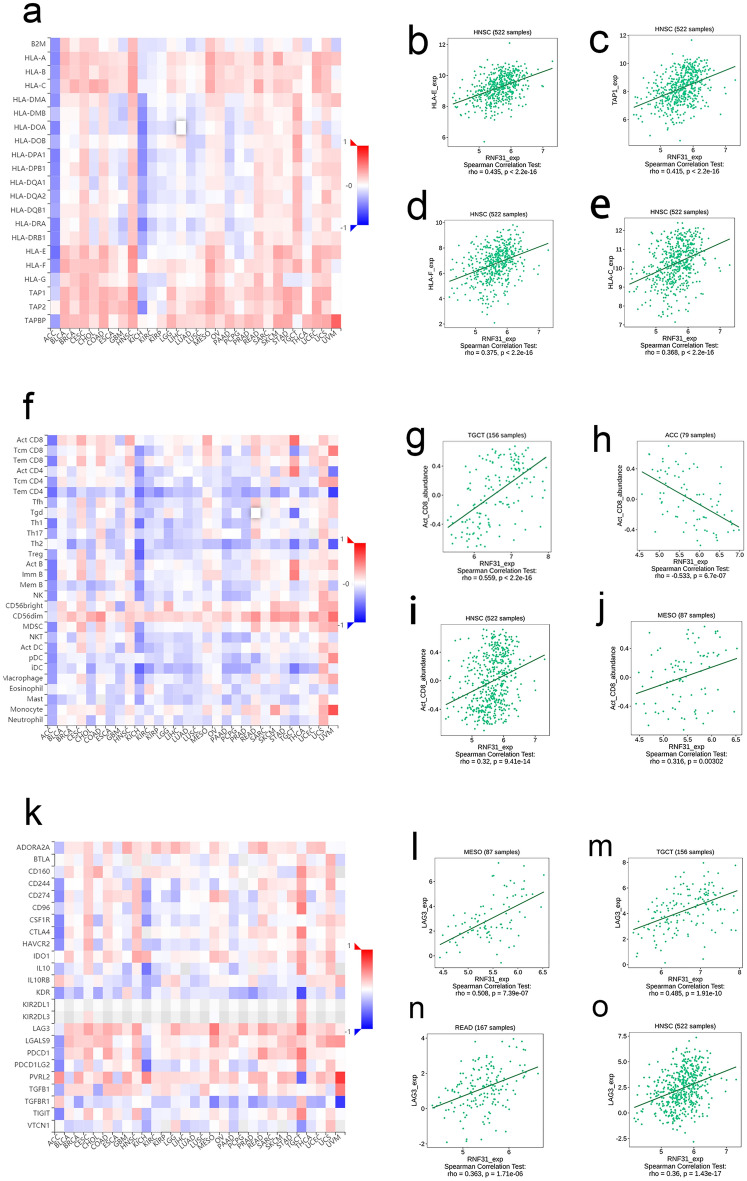
Relationship of RNF31 expression with major histocompatibility complex (MHC) molecules, tumor infiltrating lymphocytes (TILs) abundances and immunosuppressive molecules expression in human cancers. (**a**) Correlation of RNF31 and MHCs across human cancers. (**b**–**e**) RNF31 expression was positively correlated with HLA-E, TAP1, HLA-F and HLA-C in HNSC. (**f**) Correlation between RNF31 and TILs across human cancers. (**g**–**j**) RNF31 expression was related to Act CD8 in TGCT, ACC, HNSC and MESO. (**k**) Correlation between RNF31 and immunosuppressive molecules across human cancers. (**l**–**o**) RNF31 expression was related to LAG3 in MESO, TGCT, READ and HNSC. exp: expression, B2M: beta2-microglobulin, HLA: human leukocyte antigen, TAP: the transporter associated with antigen processing, Act CD8: activated CD8 T cell, Tcm CD8: central memory CD8 T cell, Tem CD8: effector memory CD8 T cell, Act CD4: activated CD4T cell, Tcm CD4: central memory CD4 T cell, Tem CD4: effector memory CD4 T cell, Tfh: T follicular helper cell, Tgd: gamma delta T cell, Th1: type 1 helper cell, Th17: type 17 helper cell, Th2: type 2 helper cell, Treg: regulatory T cell, Act B: activated B cell, Imm B: immature B cell, Mem B: memory B cell, NK: natural killer cell, CD56bright: CD56bright natural killer cell, CD56dim: CD56dim natural killer cell, MDSC: myeloid-derived suppressor cell, NKT: natural killer T cell, Act DC: activated dendritic cell, pDC: plasmacytoid dendritic cell, iDC: immature dendritic cell), Mast: mast cell.

We next evaluated the association of RNF31 with immune cell infiltration levels and immune marker gene. Our results suggested that RNF31 expression was positively associated with most of the major histocompatibility complex (MHC) molecules across human cancers except for ACC and KICH (Fig. 2a). In HNSC, for example, the top 4 MHCs were HLA-E, TAP1, HLA-F and HLA-C (Fig. 2b-e). Figure 2f shows the correlations between RNF31 expression and the abundances of tumor infiltrating lymphocytes (TILs), and the results indicated that there were significant correlations in 30 cancer types. Our results showed that RNF31 was positively correlated with the levels of CD56dim natural killer cell and CD56bright natural killer cell infiltration in multiple cancers and negatively correlated with the levels of most other infiltrating immune cells. In the case of activated CD8 T cells, the tumors with the highest correlations were TGCT, ACC, HNSC and MESO (Fig. 2g,h,i,j). For immunosuppressive molecules, there was a strong correlation in multiple cancers (Fig. 2k). Taking the immune checkpoint gene LAG3 as an example, RNF31 expression was positively correlated with LAG3 in almost all cancers. The four tumors with the strongest correlation between RNF31 and LAG3 were MESO, TGCT, READ and HNSC (Fig. 2l,m,n,o).

### Correlation between RNF31 expression and the ImmuneScore and StromalScore across cancers

**Figure 3:**
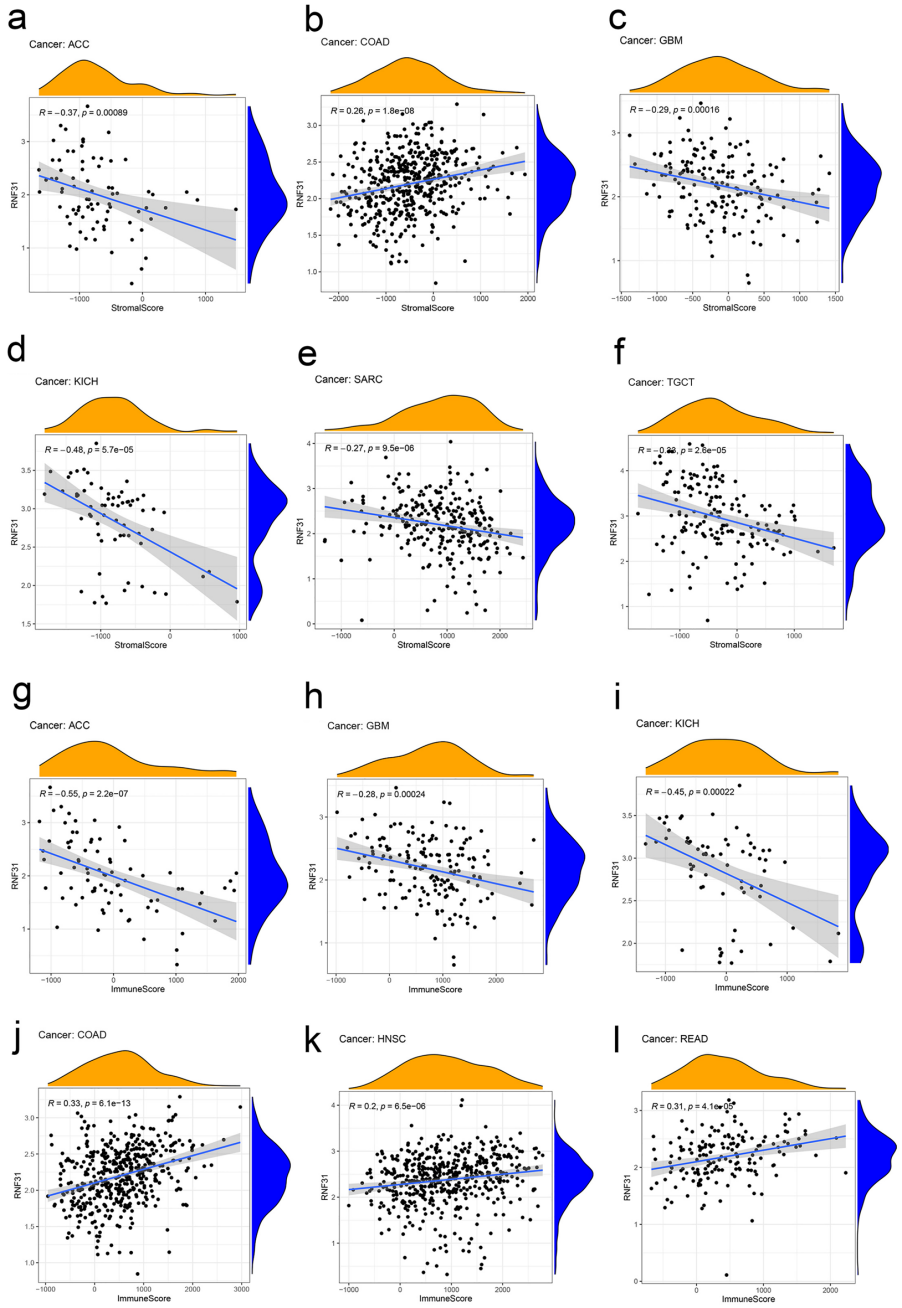
Correlation coefficients between RNF31 expression and the tumor microenvironment factors. (**a**–**f**) Correlation between RNF31 and stromal scores in ACC, COAD, GBM, KICH, SARC, and TGCT. (**g**–**l**) Correlation between RNF31 and immune scores in ACC, GBM, KICH, COAD, HNSC, and READ.

Immune cells and stromal cells in the tumor microenvironment can influence the prognosis of cancer and the survival outcomes of patients^16^. From the UCSC Xena database, we obtained 33 cancer datasets. We scored all these datasets, including the StromalScore and ImmuneScore. Then we analysed the relationship between RNF31 expression and the StromalScore and ImmuneScore. We show the most significant results in Fig. 3. There was a negative correlation between RNF31 expression and ACC, GBM, KICH, SARC and TGCT StromalScore (Fig. 3a,c,d,e,f). In contrast, we observed a positive correlation between RNF31 expression and the StromalScore in COAD (Fig. 3b). In terms of the ImmuneScore, in ACC, GBM and KICH, there was a negative correlation between RNF31 expression and score (Fig. 3g,h,i). In contrast, the ImmuneScore was positively correlated with RNF31 expression in COAD, HNSC and READ (Fig. 3j,k,l).

Based on these results, we speculate that RNF31 may affect the composition of the tumor microenvironment. For example, the expression of RNF31 was negatively correlated with stromal and immune scores in ACC. These results suggest that higher RNF31 expression is associated with higher purity of ACC tumor cells.

### Expression of RNF31 in LIHC and its effect on prognosis

**Figure 4:**
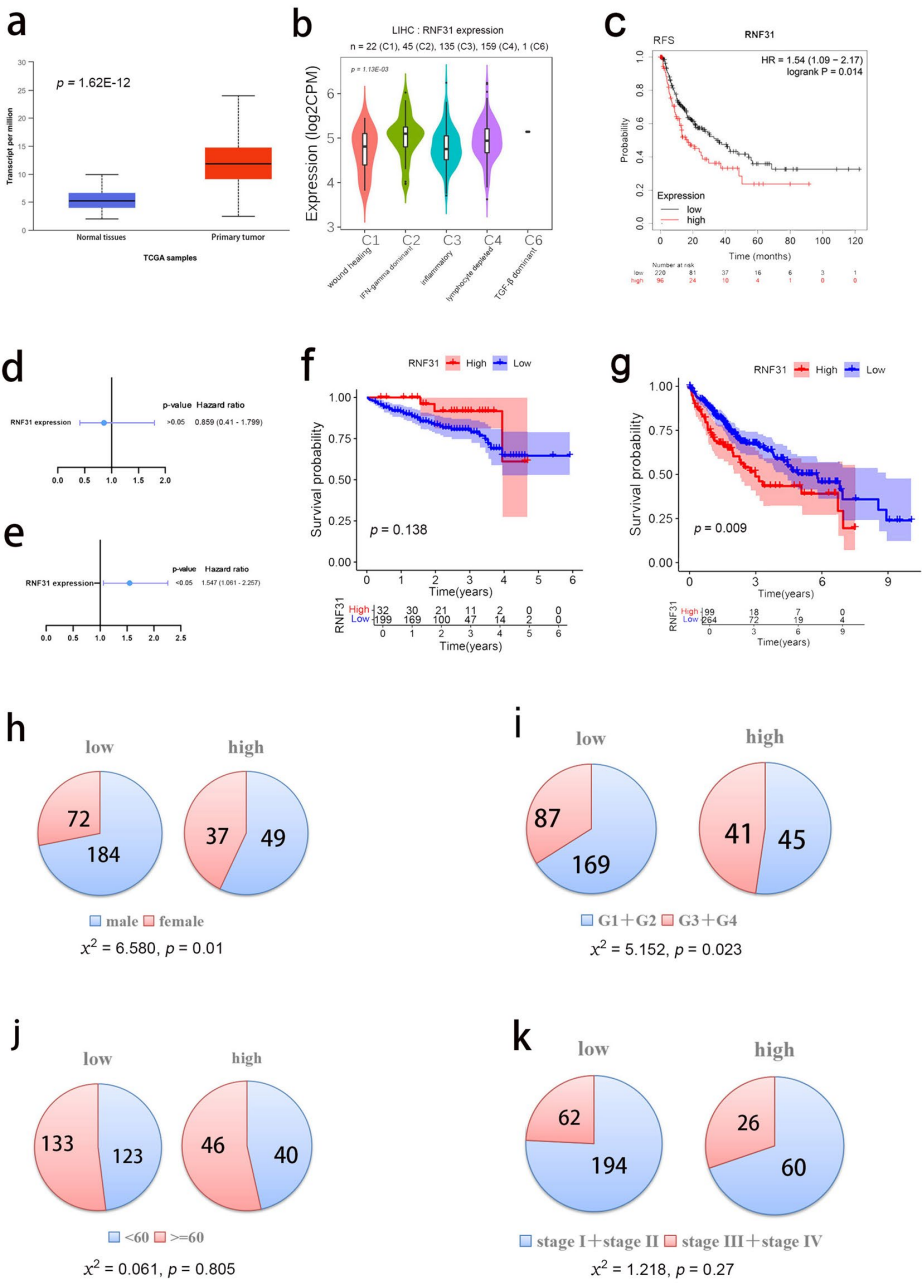
Expression of RNF31 in LIHC. (**a**) Expression of RNF31 in LIHC based on sample type. (**b**) Expression levels of RNF31 in different immune subtypes of LIHC. (**c**) Recurrence-free survival (RFS) curves of patients with LIHC. (**d**) Impact of RNF31 on OS in ICGC LIHC samples (COX regression). (**e**) Impact of RNF31 on OS in TCGA LIHC samples (COX regression). (**f**) OS curve based on ICGC LIHC samples. (**g**) OS curve based on TCGA LIHC samples. (**h**–**k**) Distribution of gender, tumor grade, age and tumor stages in high and low groups and chi-square test results (patients with RNF31 expression below the third quartile were assigned to the “low” group, and patients with expression above the third quartile were assigned to the “high” group, G1: grade1, G2: grade2, G3: grade3, G4: grade4).

We analysed the mRNA level of RNF31 using TCGA-LIHC samples in the UALCAN database. We found that RNF31 expression was significantly higher in cancer samples than in normal samples (Fig. 4a). In addition, we explored the role of RNF31 expression in immune subtypes in LIHC using the TISIDB website. RNF31 showed higher expression in subtype C2 (IFN-gamma dominant) than in the other subtypes (Fig. 4b).

Next, the Kaplan–Meier plotter database was used to evaluate the prognostic value of RNF31 in patients with LIHC. As shown in Fig. 4c, higher RNF31 expression predicted worse RFS. We analysed the impact of RNF31 on OS based on the ICGC-LIHC and TCGA-LIHC dataset (Fig. 4d,e,f,g). COX regression and Kaplan–Meier analyses suggested an association between high RNF31 expression and poor OS in TCGA-LIHC dataset (Fig. 4e,g). So, we explored the dataset further. We divided TCGA-LIHC patients into two groups based on the third quartile of RNF31 expression (patients with RNF31 expression below the third quartile were assigned to the “low” group, and patients with expression above the third quartile were assigned to the “high” group). We analysed the distribution of sex, tumor grade, age and tumor stages in the two groups (Fig. 4h,I,j,k). According to the results of the chi-square test, the proportion of low RNF31 expression in male patients was higher than that in female patients and the proportion of low RNF31 expression in grade G1 + G2 was higher than that in grade G3 + G4 (*p* < 0.05). So, we speculate that these two factors may influence the prognosis of patients together with RNF31.

### RNF31 expression is correlated with the immune infiltration level in LIHC

**Figure 5:**
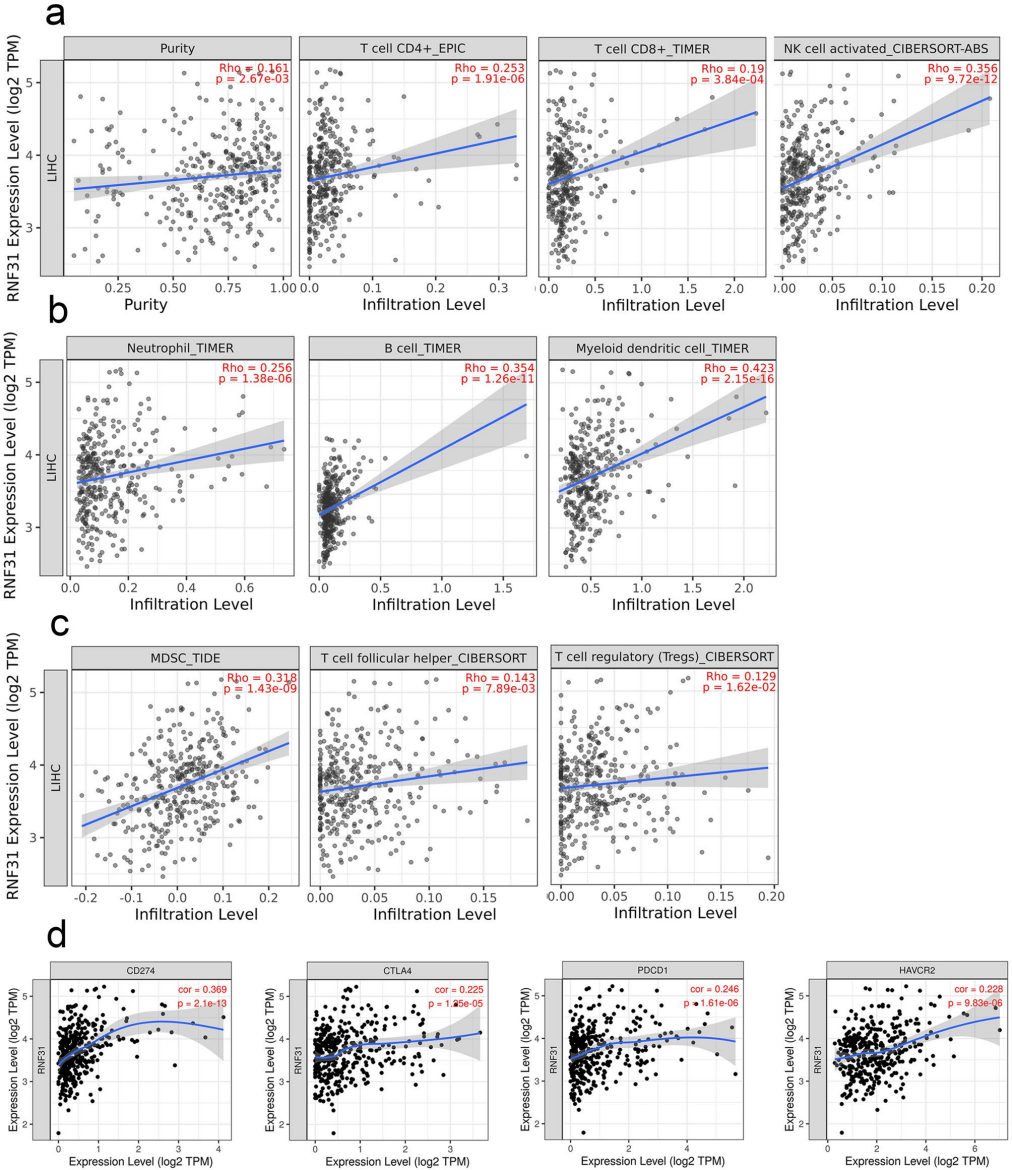
Correlation of RNF31 expression with the levels of infiltrating immune cells in LIHC. (**a**) RNF31 expression was significantly positively correlated with the levels of infiltrating CD4 + T cells, CD8 + T cells and activated NK cells. (**b**) RNF31 expression was significantly positively correlated with the levels of infiltrating neutrophils, B cells and myeloid dendritic cells. (**c**) RNF31 expression was significantly positively correlated with the levels of infiltrating myeloid-derived suppressor cells (MDSCs), follicular T cells and regulatory T cells (Tregs). (**d**) RNF31 was positively correlated with CD274, CTLA4, PDCD1 and HAVCR2 expression in LIHC.

We then investigated whether RNF31 expression was correlated with immune infiltration levels in LIHC. The results showed that RNF31 expression was significantly correlated with the levels of infiltrating CD4 + T cells, CD8 + T cells and activated NK cells (Fig. 5a). It is worth noting that there was a stronger association between RNF31 expression and the levels of infiltrating neutrophils, B cells and myeloid dendritic cells (Fig. 5b). During the analysis, we also found a significant positive correlation between RNF31 and the levels of infiltrating myeloid-derived suppressor cells (MDSCs), follicular helper T cells and regulatory T cells (Tregs; Fig. 5c). Considering that there was also a positive correlation trend between RNF31 and CD8 + T cells, we next explored the relationship of RNF31 with immune checkpoint genes in LIHC. The results showed that there was a significant positive correlation between RNF31 and several immune checkpoint genes (Fig. 5d, supplementary Figures S1), such as CD274 (PD-L1, programmed death ligand 1), CTLA4 (cytotoxic T lymphocyte antigen 4), PDCD1 (PD-1, programmed cell death-1) and HAVCR2 (hepatitis A virus cellular receptor 2).

Based on these results, we conclude that RNF31 is related to the infiltration of many kinds of immune cells and may impact immune activities in LIHC.

### Identification of RNF31-related signaling pathways in LIHC by GSEA

**Figure 6:**
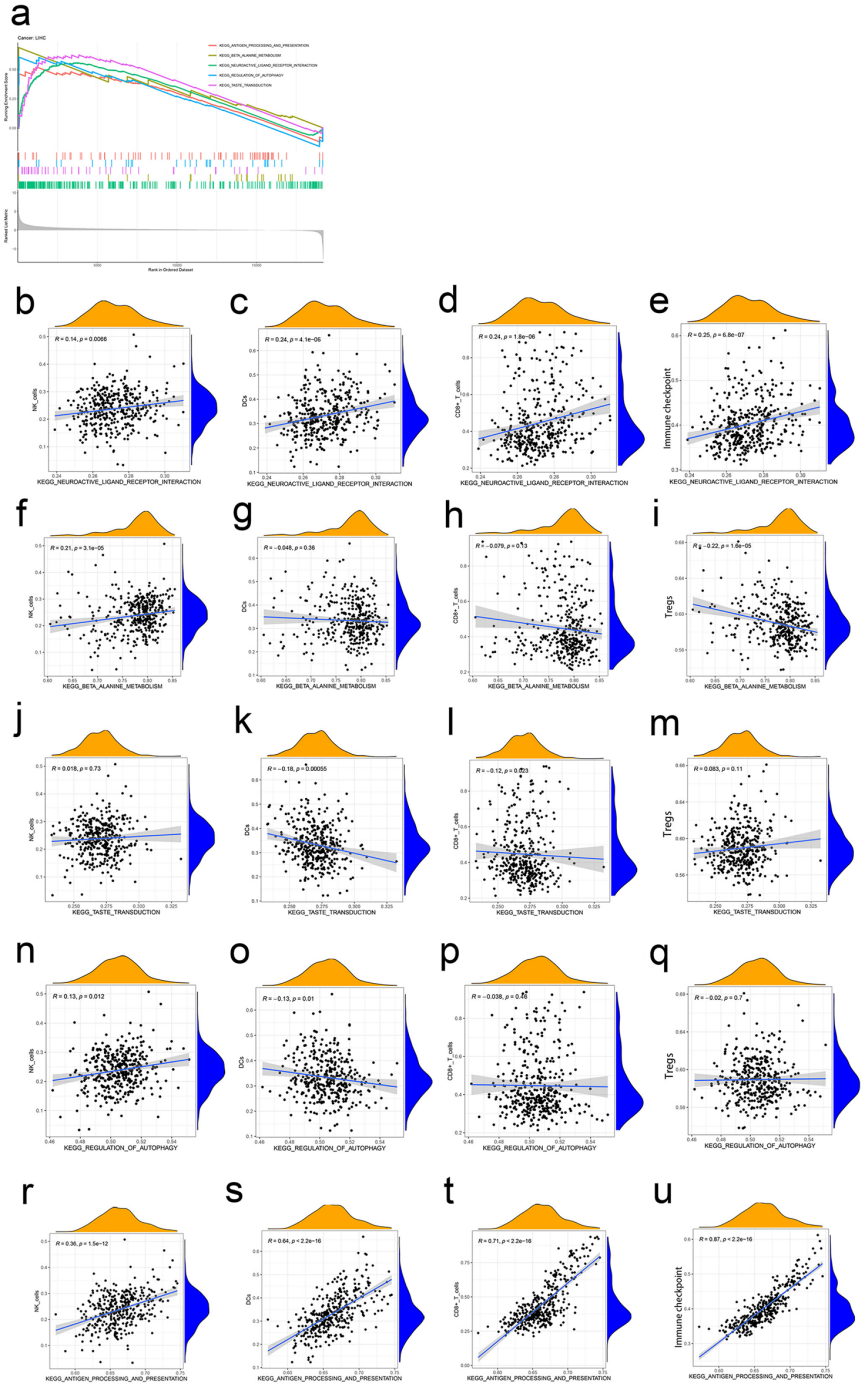
GSEA in the TCGA-LIHC dataset. (**a**) GSEA in LIHC. (**b-e**) Correlation between the “KEGG neuroactive ligand receptor interaction" pathway and NK cells, DCs, CD8 + T cells and immune checkpoint. (**f**–**i**) Correlation between the "KEGG βalanine metabolism" pathway and NK cells, DCs, CD8 + T cells and Tregs. (**j**–**m**) Correlation between the “KEGG taste transduction” pathway and NK cells, DCs, CD8 + T cells and Tregs. (**n**–**q**) Correlation between the “KEGG regulation of autophagy" pathway and NK cells, DCs, CD8 + T cells and Tregs. (**r**–**u**) Correlation between the "KEGG antigen processing and presentation" pathway and NK cells, DCs, CD8 + T cells and immune checkpoint.

On the basis of the TCGA LIHC dataset, we explored the function of RNF31 and its related signal transduction pathway through GSEA (gene set enrichment analysis). The top 5 significantly enriched signaling pathways were “KEGG neuroactive ligand receptor interaction”, “KEGG beta alanine metabolism”, “KEGG taste transduction”, “KEGG regulation of autophagy”, and “KEGG antigen processing and presentation”. KEGG is a manually curated database resource that integrates various biological objects including systems, genomic, chemical, and health information^17^. All the five KEGG pathways were enriched in the samples with high expression of RNF31 (Fig. 6a). We next performed ssGSEA (single sample GSEA) on these 5 pathways as well as immune cells (e.g., NK cells) and immune function-related factors (e.g., immune checkpoint) with the dataset and explored their relationships. For the “KEGG neuroactive ligand receptor interaction” pathway, there was a positive correlation with NK cells, DCs (dendritic cells), and CD8 + T cells (Fig. 6b,c,d). At the same time, it should be noted that there was also a close positive correlation with the immune checkpoint (Fig. 6e). The “KEGG beta alanine metabolism” pathway was positively correlated with NK cells and negatively correlated with Tregs (Fig. 6f,g,h,i). The “KEGG taste transduction” pathway was negatively correlated with DCs and CD8 + T cells (Fig. 6j,k,l,m). The “KEGG regulation of autophagy” pathway was positively correlated with NK cells and negatively correlated with DCs (Fig. 6n,o,p,q). In terms of “KEGG antigen processing and presentation”, it showed a strong positive correlation with NK cells, DCs, and CD8 + T cells (Fig. 6r,s,t). We also noted its close connection with the immune checkpoint (Fig. 6u).

### RNF31 was correlated with TNF and IFN-γ pathways in LIHC

**Figure 7:**
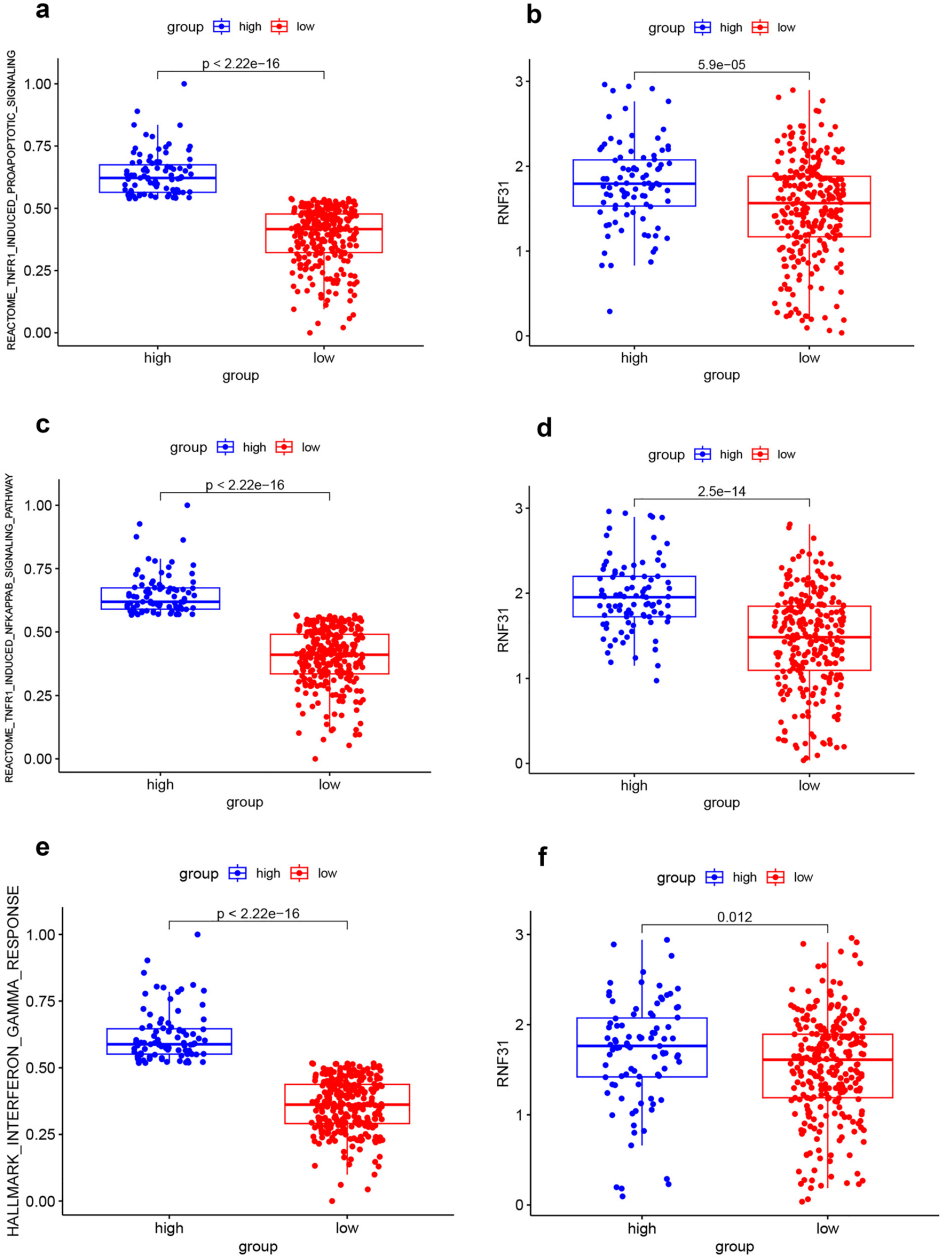
The relationship between TNF and IFN-γ pathways and RNF31 expression. (**a**–**b**) Differences in "REACTOME_TNFR1_INDUCED_PROAPOPTOTIC_SIGNALING" pathway ssGSEA scores and RNF31 expression between the two groups (patients with pathway ssGSEA scores below the third quartile were assigned to the “low” group, and patients with scores above the third quartile were assigned to the “high” group). (**c**–**d**) Differences in "REACTOME_TNFR1_INDUCED_NFKAPPAB_SIGNALING_PATHWAY" pathway ssGSEA scores and RNF31 expression between the two groups (patients with pathway ssGSEA scores below the third quartile were assigned to the “low” group, and patients with scores above the third quartile were assigned to the “high” group). (**e**–**f**) Differences in "HALLMARK_INTERFERON_GAMMA_RESPONSE" pathway ssGSEA scores and RNF31 expression between the two groups (patients with pathway ssGSEA scores below the third quartile were assigned to the “low” group, and patients with scores above the third quartile were assigned to the “high” group).

Cytotoxic T cells induce death of target cells via different processes, including the release of TNF and IFN-γ. Here, we explored whether there was an association between RNF31 and these effector mechanisms. Engagement of the TNF receptor triggers several signaling pathways, including the NFκB signaling and apoptosis induction signaling. For “REACTOME_TNFR1_INDUCED_PROAPOPTOTIC” signaling pathway. We performed ssGSEA scores for this signaling pathway in the TCGA-LIHC dataset. Next, we divided patients into two groups with the third quartile of ssGSEA scores for this signaling pathway as the cut-off (patients with scores lower than the third quartile were in the "low " group, and patients with scores higher than the third quartile were in the "high" group). We compared the differences in ssGSEA scores and RNF31 expression between the two groups. There was a significant difference in ssGSEA scores between the two groups (Fig. 7a). RNF31 expression was higher in patients with higher pathway scores (*p* < 0.05*,* Fig. 7b). We performed similar analysis for the “REACTOME_TNFR1_INDUCED_NFKAPPAB_SIGNALING_PATHWAY” and “HALLMARK_INTERFERON_GAMMA_RESPONSE” signaling pathways. For the “REACTOME_TNFR1_INDUCED_NFKAPPAB_SIGNALING_PATHWAY”. There was a significant difference in the pathway scores between the two groups (Fig. 7c). RNF31 expression was higher in the patients with higher pathway scores (*p* < 0.05*,* Fig. 7d). For the “HALLMARK_INTERFERON_GAMMA_RESPONSE”, there was a significant difference in pathway scores between the two groups (Fig. 7e). RNF31 expression was higher in the patient group with higher pathway scores (*p* < 0.05*,* Fig. 7f*).*

In summary, RNF31 was correlated with TNF and IFN-γ pathways, suggesting RNF31 may affect tumor immunity by participating in these cytokine-related pathways.

## Discussion

RNF31 contributes to the regulation of important physiological and pathological processes, including apoptosis and inflammation, proliferative capacity and protein stability^18,19^. These processes are relevant to cancer progression. Previously published literature has also revealed key roles for RNF31 in cancers. RNF31 has been reported to be critical in Epstein‒Barr virus (EBV)-mediated tumorigenesis^20^. RNF31 attenuates apoptotic cell death induced by the chemotherapy drug cisplatin^21^. In human gastric tumors, RNF31-mediated atypical ubiquitination stabilizes Forkhead box P3 and thereby stimulates regulatory T-cell function, thus promoting tumor progression^22^. RNF31 has been reported to be significantly increased in prostate cancer (PCa) and has been associated with some malignant behaviors, suggesting that RNF31 plays an oncogenic role in PCa progression. RNF31 silencing inhibits PCa cell proliferation and metastasis in vitro and in vivo^23^. RNF31 promotes p53 degradation in breast cancer cells, whereas depletion of RNF31 in breast cancer cells results in cell cycle arrest and increased cisplatin-induced apoptosis^24^. These studies reveal a significant inverse correlation between RNF31 expression and survival time, suggesting that RNF31 may be used as a pro-oncogenic factor and a prognostic marker in cancers.

In this report, we first evaluated the expression of RNF31 across cancers in the TIMER database and found that RNF31 expression was significantly upregulated in multiple cancer cells compared with normal tissues. This is consistent with previous studies^25^. Here, we found that high RNF31 mRNA expression was related to the occurrence and development of LIHC. The expression of RNF31 in cancer cells was higher than that in normal tissues. High expression of RNF31 was closely related to the poor prognosis of LIHC. Based on the TCGA LIHC dataset, we plotted a patient survival curve according to the RNF31 expression level and analysed the distribution patient clinical characteristics. There was a significant difference in the proportion of male and female patients between the high and low RNF31 expression groups. The proportion of male patients in the low expression group was higher. We thus hypothesized that sex may be a factor affecting RNF31 expression in cancer tissues. At the same time, the proportion of patients with grade 1 or grade 2 in the low RNF31 expression group was higher than that in the high group. Grade 1 and grade 2 are relatively well-differentiated cancer tissues, and the degree of malignancy is relatively low. Patients with low RNF31 expression had a better prognosis. The results further suggest the possibility of RNF31 as a prognostic factor in LIHC.

Another key finding of the present study is that RNF31 is related to immunity. There are many mechanisms that protect tumor cells from being cleared by immune cells, including insufficient antigenicity^26^, high expression of PD-L1^27^, exclusion of dendritic cells while attracting T regulatory cells^28^ and suppressive myeloid populations^29,30^. In particular, RNF31 is significantly related to the level of immune infiltration in LIHC. According to the analysis of the TISIDB data website, the expression of RNF31 in LIHC was different in different immune subtypes, and the expression of RNF31 in the IFN-γ subtype was higher than that in other subtypes. The degrees of immune infiltration is associated with prognosis and tumor progression^31^. Infiltrating immune cells, such as CD8 + T cells, B cells, CD4 + T cells, and NK cells, secrete various factors that influence the tumor microenvironment, regulate tumor behaviors, and have anticancer properties. We found a positive correlation between RNF31 and all four types of immune cells mentioned above. Immune checkpoint genes play an important role in cancers. For example, the unusually high expression of PD-L1 on tumor cells and antigen-presenting cells in the tumor microenvironment mediates tumor immune escape^32^. Immune checkpoint inhibitors are being increasingly used to treat several types of cancer^33^. Here, we found a strong positive correlation between immune checkpoint genes and RNF31 in LIHC, suggesting that RNF31 may affect T cell cytotoxicity. We performed GSEA with the TCGA LIHC dataset and obtained the top 5 active pathways in the group of patients with high RNF31 expression. Next, we calculated ssGSEA scores for immune cells in the samples and analysed the relationship between these 5 active pathways and immune cell infiltration. For the “KEGG antigen processing and presentation” pathway, the most significant correlation was with the levels of infiltrating immunogenic CD8 + T cells, NK cells and DC cells. In addition, RNF31 was positively correlated with the levels of infiltrating immunosuppressive cells such as MDSCs and Tregs, both of which protect cancer cells from damage from the patient's immune system^34,35^. These findings indicate that RNF31 plays an important role in regulating tumor immunity and therefore affects the prognosis of LIHC. These results support the possibility of RNF31 as an immunotherapeutic target.

In addition, that “KEGG pathway regulation of autophagy” was found to be enriched in the GSEA. Autophagy is considered a distinct type of programmed cell death. Due to its involvement in cell death, autophagy may be a tumor-suppressive mechanism^36^. However, there is evidence that autophagy actually supports tumorigenesis in some contexts and may promote tumor growth and cancer cell survival in established tumors^37,38^. It may play a dual role in promoting and inhibiting tumor processes depending on the type or context of the tumor. RNF31 knockdown inhibits the initiation of autophagy^39^. Other reports have indicated that autophagy can promote tumor immune escape^40,41^. In our GSEA, we found that the autophagy pathway was active in patients with high RNF31 expression. Therefore, we speculated that RNF31 may affect tumor immunity by affecting the expression of autophagy-related molecules in LIHC. However, this hypothesis needs to be investigated by follow-up experiments.

The production of the cytokines TNF and IFN-γ is an antitumor activity performed by CD8 + T and NK cells^42^. In addition, LUBAC plays a key role in TNF signaling^43^. RNF31 is an important subunit of LUBAC. Therefore, we hypothesized that RNF31 may affect tumor immunity by participating in these cytokine-related pathways. We calculated TNF and IFN-γ pathway ssGSEA scores in TCGA LIHC samples separately. We divided patients into two groups with the third quartile of ssGSEA scores as the cut-off (patients with scores lower than the third quartile were in the "low " group, and patients with scores higher than the third quartile were in the "high" group). We found significant differences in RNF31 expression between the two groups. This finding preliminarily supports our hypothesis that RNF31 is involved in the effects of TNF and IFN-γ. TNF may induce different physiological processes, including NF-κB pro-survival activities and stimulation of cell death^44^. RNF31 deficiency not only impairs TNF-induced NF-κB activation but also significantly increases apoptosis^18^. In this study, we found that RNF31 was associated with both of these effector processes. Small molecule RNF31 inhibitors sensitize colon cancer organoids to TNF-mediated death^14^. However, whether the same effect can be seen in LIHC requires further investigation.

## Conclusions

In this study we investigated the role of RNF31 in LIHC. High expression of RNF31 is associated with poor prognosis. This suggests that RNF31 may serve as a prognostic marker for LIHC. RNF31 correlates with the levels of infiltrating immune cells. At the same time, RNF31 is strongly associated with immune checkpoint genes expression in LIHC, suggesting the possibility of RNF31 as a target for immunotherapy.

## Materials and methods

### Data collection

The Cancer Genome Atlas (TCGA) RNA-seq data for 33 cancers and LIHC survival data were downloaded from the University of California Santa Cruz (UCSC) Xena browser (https://xenabrowser.net/datapages/). ICGC LIHC data was downloaded from International Cancer Genome Consortium (ICGC) database (https://dcc.icgc.org/releases/current/Projects/LIRI-JP).

### RNF31 expression and prognostic value across cancers

The transcriptional signature of RNF31 across cancers was analysed based on the Tumor Immune Estimation Resource (TIMER, https://cistrome.shinyapps.io/timer/) database and The University of ALabama at Birmingham CANcer data analysis Portal (UALCAN, http://ualcan.path.uab.edu/) network. The difference of RNF31 expression across immune and molecular subtypes was analysed using the TISIDB (http://cis.hku.hk/TISIDB/). The relationship of RNF31 mRNA expression with relapse-free survival (RFS) in LIHC was determined via analysis of Kaplan–Meier Plotter (http://kmplot.com/analysis/index.php?p=service).

### Cox regression analysis and Kaplan–Meier survival analysis

Cox regression analysis was used to evaluate the relationship between RNF31 expression and overall survival (OS) of patients using the ICGC-LIHC and TCGA-LIHC data. The Kaplan–Meier method was used to assess the difference between “high” and “low” groups based on the best separation of RNF31 expression, employing R packages of “survminer” and “survival”. The “surv-cutpoint” function in the survminer R package was performed to search the best split by verifying all potential cut points.

### GSEA

Gene set enrichment analysis (GSEA) was performed to explore potential pathways associated with RNF31 using the Kyoto Encyclopedia of Genes and Genomes (KEGG) terms and the R packages "org.Hs.eg.db", "clusterProfiler" and "enrichplot".

### ssGSEA

Single-sample GSEA (ssGSEA) was used to calculate the scores of immune cells, immune function and related pathways in LIHC samples using the R packages "GSEABase" and "GSVA". The gene sets of immune cells used for ssGSEA is provided in the supplementary Table S1.

### Correlation between RNF31 and immune cells

Relationships between the abundances of tumor-infiltrating lymphocytes (TILs) and the levels of, immunomodulators (MHC molecules and immunosuppressive molecules) and RNF31 expression were investigated using the TISIDB database. R package “ESTIMATE” was used to calculate the StromalScore and ImmuneScore based on TCGA datasets. We then used a Spearman correlation analysis to analyse the relationship between RNF31 and StromalScore and ImmuneScore. The TIMER database was used to analyse the correlation of RNF31 with the levels of infiltrating immune cells and the expression of immune checkpoint genes in LIHC.

### Molecular signatures database

We downloaded collections of signal pathway-related genes from the Molecular Signatures Database (MSigDBhttp://www.gsea-msigdb.org/gsea/msigdb/), including five KEGG (Kyoto Encyclopedia of Genes and Genomes) pathways derived from GSEA (KEGG_ANTIGEN_PROCESSING_AND_PRESENTATION, KEGG_BETA_ALANINE_METABOLISM, KEGG_NEUROACTIVE_LIGAND_RECEPTOR_INTERACTION, KEGG_REGULATION_OF_AUTOPHAGY, KEGG_TASTE_TRANSDUCTION) and TNF and IFN-γ signaling pathways (REACTOME_TNFR1_INDUCED_PROAPOPTOTIC_SIGNALING, REACTOME_TNFR1_INDUCED_NFKAPPAB_SIGNALING_PATHWAY, and HALLMARK_INTERFERON_GAMMA_RESPONSE).

### Statistical analysis

Statistical analyses were performed using SPSS v23 and R software (version 4.1.2). The boxplots were generated using the R package "ggpub". The scatter plots were generated using the R package "ggplot2", "ggpubr", and "ggExtra". The forest plots were generated using GraphPad Prism 9 (GraphPad Software). We analysed the difference in RNF31 expression between patients with different sexes, tumor grades, ages and tumor stages using a chi-square test. *P* < 0.05 was considered statistically significant.

## Supplementary Information

Supplementary Information 1.

Supplementary Information 2.

## Data Availability

The datasets presented in this study can be found in online repositories. The mRNA expression data of 33 types of cancers and corresponding clinical information were acquired from The Cancer Genome Atlas through the UCSC Cancer Genome Browser (https://xenabrowser.net/datapages/,accessed March 2022). ICGC LIHC data was downloaded from International Cancer Genome Consortium (ICGC) database (https://dcc.icgc.org/releases/current/Projects/LIRI-JP, accessed March 2023). Further inquiries can be directed to the corresponding authors.
