# Supplementary material for: High expression of RNF31 is associated with tumor immune cell infiltration and leads to poor prognosis in liver hepatocellular carcinoma
Source: Sci Rep. 2023 Apr 28;13:6957. doi: 10.1038/s41598-023-32692-4 (PMC10147728; doi:10.1038/s41598-023-32692-4)
Supplement: Supplementary file 1 — Supplementary Information 1. [file 41598_2023_32692_MOESM1_ESM.docx]

High expression of RNF31 is associated with tumor immune cell infiltration and leads to poor prognosis in liver hepatocellular carcinoma

Guifu Xi^1†^, Runfen Cheng^2†^, Leiting Liang^1^, Na Che^1^, Yalei Wang^2^, Nan Zhao^1^, Xiaohui Liang^1^, Bing Shao^2^, Xiulan Zhao^1*^, Danfang Zhang^1*^

† These authors contributed equally to this work and share first authorship

1 Department of Pathology, Tianjin Medical University, Tianjin 300070, China

2 Tianjin Medical University Cancer Institute and Hospital, National Clinical Research Center for Cancer, Key Laboratory of Cancer Prevention and Therapy, Tianjin’s Clinical Research Center for Cancer, Tianjin 300060, China

*Correspondence:

Danfang Zhang, E-mail: [zhangdf@tmu.edu.cn](mailto:zhangdf@tmu.edu.cn).

Xiulan Zhao, E-mail: zhaoxiulan@tmu.edu.cn.


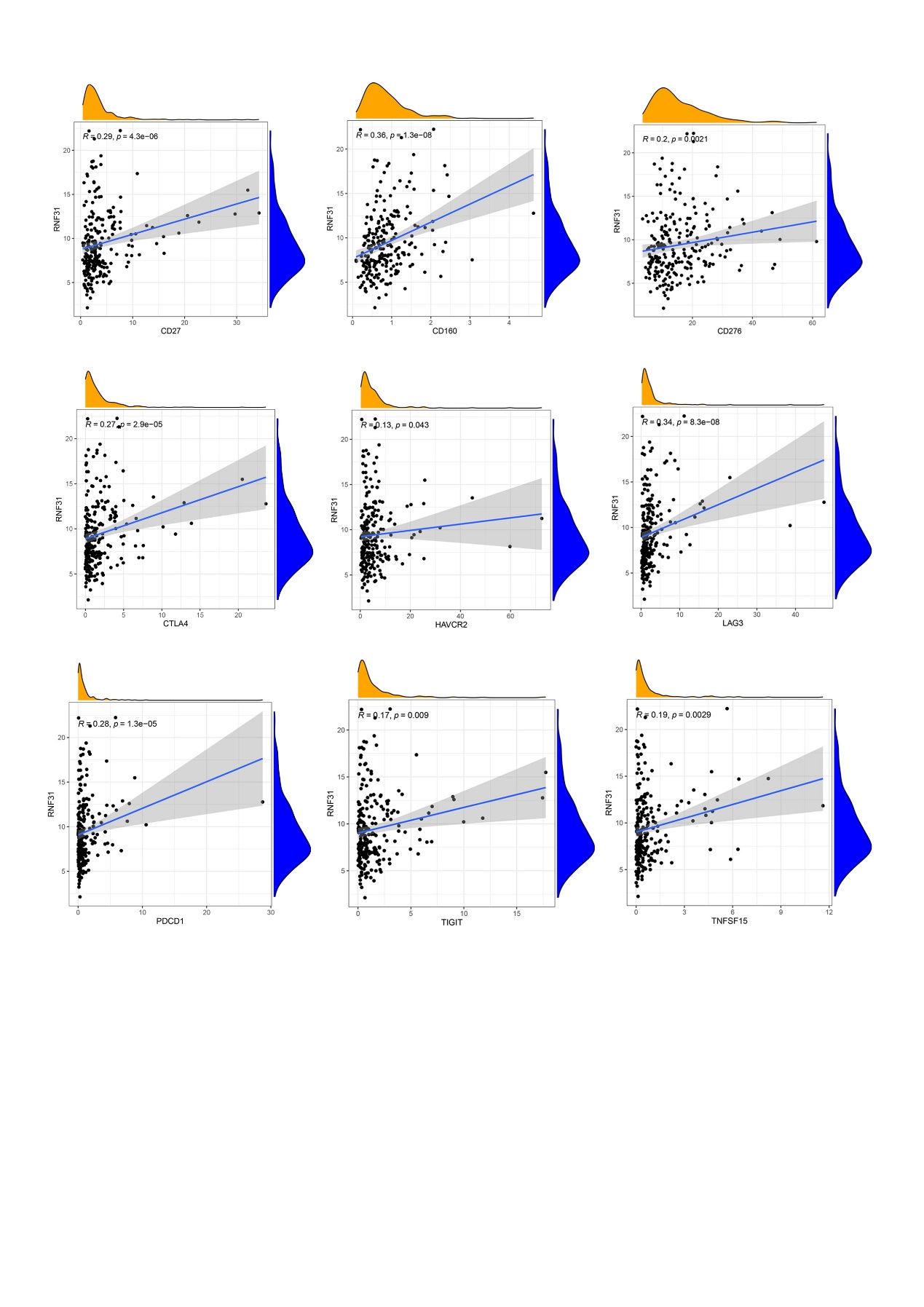


**Supplementary** **Figure** **S1**| A significant positive correlation between RNF31 and immune checkpoint genes.
