# Supplementary material for: High expression of RNF31 is associated with tumor immune cell infiltration and leads to poor prognosis in liver hepatocellular carcinoma
Source: Sci Rep. 2023 Apr 28;13:6957. doi: 10.1038/s41598-023-32692-4 (PMC10147728; doi:10.1038/s41598-023-32692-4)
Supplement: Supplementary file 2 — Supplementary Information 2. [file 41598_2023_32692_MOESM2_ESM.docx]

**High expression of RNF31 is associated with tumor immune cell infiltration and leads to poor prognosis in liver hepatocellular carcinoma**

**Guifu Xi^1†^, Runfen Cheng^2†^, Leiting Liang^1^, Na Che^1^, Yalei Wang^2^, Nan Zhao^1^, Xiaohui Liang^1^, Bing Shao^2^, Xiulan Zhao^1*^, Danfang Zhang^1*^**

† These authors contributed equally to this work and share first authorship

1 Department of Pathology, Tianjin Medical University, Tianjin 300070, China

2 Tianjin Medical University Cancer Institute and Hospital, National Clinical Research Center for Cancer, Key Laboratory of Cancer Prevention and Therapy, Tianjin’s Clinical Research Center for Cancer, Tianjin 300060, China

***Correspondence:**

Danfang Zhang, E-mail: [zhangdf@tmu.edu.cn](mailto:zhangdf@tmu.edu.cn).

Xiulan Zhao, E-mail: zhaoxiulan@tmu.edu.cn.

| 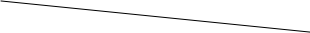 | gene set |
| --- | --- |
| NK cells | KLRC1、KLRF1 |
| DCs (dendritic cells) | CCL17、CCL22、CD209、CCL13 |
| Immune checkpoint | IDO1、LAG3、CTLA4、TNFRSF9、ICOS、CD80、 PDCD1LG2、TIGIT、CD70、TNFSF9、ICOSLG、 KIR3DL1、CD86、PDCD1、LAIR1、TNFRSF8、 TNFSF15、TNFRSF14、IDO2、CD276、CD40、 TNFRSF4、TNFSF14、HHLA2、CD244、CD274、 HAVCR2、CD27、BTLA、LGALS9、TMIGD2、CD28 、CD48、TNFRSF25、CD40LG、ADORA2A、VTCN1 、CD160、CD44、TNFSF18、TNFRSF18、BTNL2 、C10orf54、CD200R1、TNFSF4、CD200、NRP1 |
| Tregs | IL12RB2、TMPRSS6、CTSC、LAPTM4B、TFRC、 RNF145、NETO2、ADAT2、CHST2、CTLA4、 NFE2L3、LIMA1、IL1R2、ICOS、HSDL2、 HTATIP2、FKBP1A、TIGIT、CCR8、LTA、 SLC35F2、IL21R、AHCYL1、SOCS2、ETV7、 BCL2L1、RRAGB、ACSL4、CHRNA6、BATF、LAX1 、ADPRH、TNFRSF4、ANKRD10、CD274、CASP1 、LY75、NPTN、SSTR3、GRSF1、CSF2RB、 TMEM184C、NDFIP2、ZBTB38、ERI1、TRAF3、 NAB1、HS3ST3B1、LAYN、JAK1、VDR、LEPROT 、GCNT1、PTPRJ、IKZF2、CSF1、ENTPD1、 TNFRSF18、METTL7A、KSR1、SSH1、CADM1、 IL1R1、ACP5、CHST7、THADA、CD177、NFAT5 、ZNF282、MAGEH1 |
| CD8+ T cells | CD8A |

**Supplementary** **Table** **S1|** The immune cells gene set used for ssGSEA.
